# Editorial: Lifestyle modification strategies as first line of chronic disease management

**DOI:** 10.3389/fphys.2023.1204581

**Published:** 2023-05-10

**Authors:** Sechang Oh, Eonho Kim, Junichi Shoda

**Affiliations:** ^1^ Faculty of Rehabilitation, R. Professional University of Rehabilitation, Tsuchiura, Japan; ^2^ Department of Physical Education, Dongguk University, Seoul, South Korea; ^3^ Faculty of Medicine, University of Tsukuba, Tsukuba, Japan

**Keywords:** augmented reality, sarcopenia, NAFLD, Parkinson’ disease, metabolically healthy obese

## Introduction

Chronic diseases have a significant impact on life expectancy, quality of life and healthcare costs (Kaplan, 2003). The management and prevention of chronic diseases through lifestyle modification strategies involving changes in lifestyle, physical activity and healthy dietary consumption is an essential area of public health (Dalle Grave et al., 2010). However, there is a lack of awareness and practice of lifestyle modification in the general population and limitations in accounting for individual differences and social factors. This editorial address five articles published in the Research Topic ‘Lifestyle Modification Strategies as First Line of Chronic Disease Management’, which explores the potential of lifestyle modification strategies in the management and prevention of chronic diseases. The editorial highlights healthy lifestyle modifications and the importance of addressing chronic diseases, with a focus on tailored strategies to prevent and effectively manage these conditions.

## Healthy lifestyle modification

Wingenbach and Zana investigated the potential of encouraging physical activity and social activities through augmented reality games such as Pokémon Go. They found that an increase in physical activity and social relationships related to playing Pokémon Go augmented participants’ quality of life, providing empirical evidence that augmented reality games can foster social connectedness and overall wellbeing. However, the limitations of these games include the need for ongoing engagement and the potential for overuse or addiction. Despite these challenges, the findings emphasize the importance of integrated programs that encourage lifestyle changes to reduce the risk of chronic diseases and improve mental health by promoting physical activities and social symbiosis.

## Prevention and treatment of chronic diseases

Obesity is a major contributing risk factor in various chronic diseases. Su et al. identified a subgroup of metabolically healthy obese (MHO) individuals who seemed healthy but still faced an increased risk of developing metabolic disorders and cardiovascular diseases. This review article emphasized the critical role of physical activity in preventing and treating chronic diseases, particularly for MHO individuals, to correct abnormal micro-metabolic pathways of the MHO, regulate metabolic homeostasis, and enhance metabolic flexibility. The study highlighted that MHO individuals may have a normal metabolic profile but still have an increased risk of developing chronic health problems. The review emphasizes the need for healthcare professionals and researchers to be aware of the unique challenges faced by MHO individuals and to develop targeted intervention strategies that incorporate physical activity and other lifestyle modifications to prevent the onset of chronic disease and improve overall health. Sarcopenia, a decrease in muscle mass, strength, and function because of aging, gives rise to declined physical performance and an increased risk of chronic diseases. Kim et al. investigated a method that uses the circumference of calves as a tool for detecting sarcopenia early among healthy Korean adult men. The measurement of calf circumference was found to be an effective tool for assessing muscle mass in the diagnosis of sarcopenia, which enabled effective intervention and improved health results. By using a simple, non-invasive and cost-effective method such as calf circumference measurement, healthcare professionals and researchers can quickly and easily identify individuals at risk of sarcopenia and implement targeted interventions such as tailored exercise programs and dietary counseling. This practical approach can help individuals maintain their muscle mass, strength and overall functional capacity, ultimately improving their quality of life and reducing the burden on healthcare systems.

Non-alcoholic fatty liver disease (NAFLD) is a prevalent chronic liver disease closely related to obesity and metabolic disorders. The muscle-liver axis plays a crucial role in the development and progression of NAFLD, as skeletal muscle influences liver function through various metabolic pathways. Miura et al. observed that the progression of NAFLD in mice could be delayed with the rescue of p62/Sqtsm1 protein in skeletal muscles through the management of muscle health in mice fed with a high-fat diet. This finding highlights the importance of the muscle-liver axis in the management of NAFLD and suggests a novel strategy involving the improvement of p62/Sqtsm1 activity by exercise and dietary intervention. Exercise can improve insulin sensitivity in skeletal muscle, reduce lipid accumulation in the liver and promote the secretion of myokines that can modulate liver metabolism. These factors contribute to the prevention and management of NAFLD. By focusing on the muscle-liver axis and incorporating exercise and dietary interventions, healthcare professionals and researchers can develop more effective strategies for managing NAFLD and reducing its associated health risks. Parkinson’s disease (PD) is a progressive neurodegenerative disorder that impairs motor, cognitive, and behavioral abilities, ultimately reducing overall quality of life. Wang et al. conducted a comprehensive review of the long-term effects of various exercise interventions in healthy older adults and those with PD. The study emphasized the practical application of different exercise methods and demonstrated that tailored exercise programs can have a significant impact on reducing the symptoms and progression of PD. The results showed that these exercise programs led to positive functional and structural changes in the brain, improving exercise performance and daily functioning in participants with PD. In addition, the study highlighted the importance of individualized exercise programs that meet the unique needs of each person with PD, considering factors such as disease severity, physical ability and personal preferences.

The aforementioned studies underscore the potential of lifestyle change in preventing and managing chronic diseases. Researchers can develop evidence-based interventions that improve wellbeing and prevent chronic diseases by uncovering new diagnostic tools, identifying crucial proteins, or implementing a program of various exercise interventions. However, to optimize the outcome of any lifestyle change strategy, social determinants impacting our health need to be addressed. Medical experts need to recognize that a one-size-fits-all approach to lifestyle change cannot accommodate the vast differences in individuals. These experts need to be vigilant in personalizing the interventions to specific populations and individual needs. Therefore, providing comprehensive and personalized treatments to individual patients requires participation from medical experts and researchers.
